# Serologic Evidence of *Orientia* Infection among Rural Population, Cauca Department, Colombia

**DOI:** 10.3201/eid2902.221458

**Published:** 2023-02

**Authors:** Álvaro A. Faccini-Martínez, Carlos Ramiro Silva-Ramos, Lucas S. Blanton, Esteban Arroyave, Heidy-C. Martínez-Diaz, Paola Betancourt-Ruiz, Marylin Hidalgo, David H. Walker

**Affiliations:** Fundación Universitaria de Ciencias de la Salud, Bogotá, Colombia (Á.A. Faccini-Martínez);; Servicios y Asesorías en Infectología, Bogotá (Á.A. Faccini-Martínez);; Hospital Militar Central, Bogotá (Á.A. Faccini-Martínez);; University of Texas Medical Branch, Galveston, Texas, USA (Á.A. Faccini-Martínez, L.S. Blanton, E. Arroyave, D.H. Walker);; Pontificia Universidad Javeriana, Bogotá (C.R. Silva-Ramos, H.-C. Martínez-Diaz, P. Betancourt-Ruiz, M. Hidalgo)

**Keywords:** *Orientia*, serologic evidence, seroprevalence, bacteria, rural population, scrub typhus, acute undifferentiated febrile illness, AUFI, indirect immunofluorescence antibody assay, IFA, ELISA, zoonoses, Cauca Department, Colombia

## Abstract

We assessed serum samples collected in Cauca Department, Colombia, from 486 persons for *Orientia* seroreactivity. Overall, 13.8% showed reactive IgG by indirect immunofluorescence antibody assay and ELISA. Of those samples, 30% (20/67) were confirmed to be positive by Western blot, showing >1 reactive band to *Orientia* 56-kD or 47-kD antigens.

Scrub typhus, caused by species in the genus *Orientia*, is a reemerging mite-borne rickettsiosis and a major cause of acute undifferentiated febrile illness (AUFI) (*1*). Classically, scrub typhus was believed to be strictly endemic to the so-called tsutsugamushi triangle, which ranges from southeastern Siberia in the North to the Kamchatka Peninsula in the East, northern Australia in the South, and Pakistan in the West (*1*). However, scrub typhus outside the tsutsugamushi triangle was suggested 70 years ago because seropositivity to *O. tsutsugamushi* was found among persons from several countries in Africa (*1*).

In 2006, a confirmed case of scrub typhus outside the classical disease-endemic region was described in a traveler who visited Dubai (United Arab Emirates); the case was caused by a novel species, *Candidatus* Orientia chuto (*2*). More recently, in Latin America, autochthonous cases of scrub typhus have been reported in Chile and attributed to a new *Orientia* species, *Candidatu*s Orientia chiloensis, associated with *Herpetacarus* spp. mites, which have been recognized as novel vectors of scrub typhus in this newly recognized disease-endemic region (*3*,*4*).

In addition to the aforementioned studies in Chile, serologic surveys have been performed among military personnel stationed in the Peruvian Amazon and Honduras, demonstrating exposure to *Orientia*, as well as a possible role for this bacterium in the etiology of AUFI (*5*,*6*). Thus, to explore new geographic regions that might have *Orientia* circulation, we report serologic evidence of this rickettsial agent in Colombia.

We screened 486 serum samples collected in 4 municipalities in the Cauca Department, Colombia, during 2017 and used in a previous study (*7*) for *Orientia* seroreactivity. Cauca Department is located in southwestern Colombia, mainly in the Andean and Pacific regions plus a tiny part in the Amazonian region. The climate is warm and humid on the western slope; warm and semiarid toward the Patía basin; and temperate humid to semihumid centrally, with cold climates recorded on both sides of the Popayán (capital city) Plateau.

We tested for antibodies reactive to *O. tsutsugamushi* by indirect immunofluorescence antibody (IFA) assay and ELISA (Appendix). To further confirm the specificity of the reactive samples, we performed a Western blot assay (Appendix). 

Of 486 serum samples, 6 (1%) were seropositive for *Orientia* by IFA with an endpoint titer of 1:128 to 1:1,024, and 62 (13%) yielded positive results by ELISA (optical density cutoff >0.37). Only 1 sample was positive by both methods. Overall, 67 (13.8%) serum samples were positive by >1 screening method. Testing of 67 of the seropositive samples by Western blot showed that 20 (30%) were reactive with >1 *O. tsutsugamushi* band. A total of 8 samples were reactive to 56-kD, 5 to 47-kD, and 7 to both (Table; Figure).

**Table:** *Orientia* seropositivity in rural populations of Cauca Department, by testing method, Colombia, 2017*

Municipalities and rural localities	No. samples	No. (%) *Orientia* IgG positive samples		
		IFA	ELISA	Western blot†
Caloto	48	5 (10.4)	2 (4.2)	6 (100)‡
El Credo	35	4 (11.4)	1 (2.9)	4 (100)‡
Huellas	13	1 (1.7)	1 (1.7)	2 (100)
El Tambo	262	0	45 (17.2)	11 (24.4)
Betania	46	0	3 (6.5)	2 (66.7)
Placer	88	0	14 (15.9)	6 (42.9)
Zarzal	128	0	28 (21.9)	3 (7)
La Sierra	30	0	5 (16.7)	1 (20)
Juan Castaña	30	0	5 (16.7)	1 (20)
Santander de Quilichao	146	1 (0.7)	10 (6.8)	2 (18.2)
Lomitas Arriba	65	0	6 (9.2)	0
Lomitas Ajabo	81	1 (1.2)	4 (4.9)	2 (40)
Total	486	6 (1.2)	62 (12.8)	20 (29.9)‡


*IFA, indirect immunofluorescence antibody assay. †Denominators are IFA-positive or ELISA-positive samples. ‡One sample was positive by both IFA and ELISA.

**Figure:**
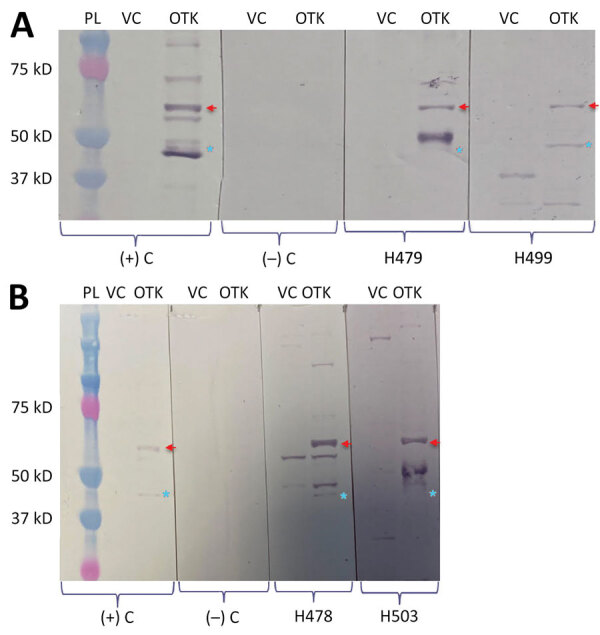
Serologic evidence of *Orientia* infection among rural population, Cauca Department, Colombia. Western blot analysis was performed using Vero cell extracts and *O. tsutsugamushi* Karp strain whole-cell antigens. Examples of some reactive samples that were applied to probe-migrating *Orientia*-specific antigens (56 kD and 47 kD) are shown: A) samples H479 and H499; B) samples H478 and H503. Red arrows indicate 56-kD protein, and blue stars indicate 47-kD protein. OTK, *O. tsutsugamushi* Karp strain; PL, protein ladder; VC, Vero cell; (‒) C, negative control; (+) C, positive control.

Our results show serologic evidence of *Orientia* exposure among the rural population of 4 municipalities from Cauca Department, Colombia. This evidence suggested circulation of this rickettsial agent in this region. 

Using 2 serologic techniques (IFA and ELISA), we found different seropositivity rates (1% for IFA and 13% for ELISA) for the same serum samples. Similar discordance has been observed for persons from Sao Tome and Principe (*8*). The difference in seropositivity rates between methods might be caused by the antigens used because our IFA used only antigens of the Karp strain, which could fail to detect antibodies stimulated by organisms of other serotypes and species. Moreover, some autochthonous cases of scrub typhus in Chile have also shown discordant serologic results (reactive IgG by IFA/negative IgG by ELISA, and vice versa) (*9*,*10*).

As briefly mentioned, the use of ELISA to detect IgG reactive against *Orientia* showed reactivity in 5.3% of convalescent-phase serum samples from febrile patients in the region of Iquitos (Peruvian Amazon) (*5*) and in 5.6% of US military personnel stationed at least 6 months in Honduras (*6*). Our study found 13% seropositivity by using the same serologic approach (IgG ELISA), which is greater than results obtained in Perú and Honduras. In addition to ELISA and IFA screening methods, we used a confirmatory test (Western blot), which demonstrated reactivity to >1 immunodominant protein (56 kD and 47 kD) of *Orientia* in 30% of the tested samples, which were positive by >1 screening method.

In conclusion, we report serologic evidence of *Orientia* in Colombia, supporting that these bacteria are probably widely distributed in Latin America. Our study also supports emerging evidence that *Orientia* spp. is no longer restricted to the tsutsugamushi triangle. Prospective studies to examine acute-phase and convalescent-phase serum samples and isolation of the agent in those with AUFI in Colombia and other countries in Latin America should be undertaken. In addition, scrub typhus should be considered as a potential diagnosis by physicians and public health officials when an eschar-associated rickettsiosis is suspected in this region.

Appendix Additional information on serologic evidence of *Orientia* infection among rural population, Cauca Department, Colombia.
