# Supplementary material for: Serologic Evidence of Orientia Infection among Rural Population, Cauca Department, Colombia
Source: Emerg Infect Dis. 2023 Feb;29(2):456–9. doi: 10.3201/eid2902.221458 (PMC9881757; doi:10.3201/eid2902.221458)
Supplement: Appendix — Additional information on serologic evidence of Orientia infection among rural population, Cauca Department, Colombia. [file 22-1458-Techapp-s1.pdf]

Article DOI: https://doi.org/10.3201/eid2902.221458

Serologic Evidence of *Orientia* Infection among Rural Population, Cauca Department, Colombia

Appendix

Methods

IFA was performed by using in-house slides coated and fixed with whole-cell antigens of *Orientia tsutsugamushi* Karp strain. Fluorescein isothiocyanate–conjugated goat antihuman IgG was used as the secondary antibody at a dilution of 1:1,600. Sera were diluted in 2-fold increments with phosphate-buffered saline, starting at a dilution of 1:128. Positive and negative serum controls were used for all reactions, which consisted of serum from a scrub typhus–confirmed patient from Asia (kindly provided by Lee Fuller from Fuller Laboratories, Fullerton, CA, USA), and a serum sample of a healthy person from Galveston, TX, USA, respectively. Detection of IgG by ELISA was performed by using the Scrub Typhus Detect IgG ELISA System (InBios International Inc., http://www.inbios.com) following the manufacturer's instructions. This ELISA uses Karp, Kato, Gilliam, and TA716 strain recombinant proteins of the 56-kD outer membrane protein.

For Western blot assay, antigens were prepared from *O. tsutsugamushi* Karp strain (cultivated in Vero cells) and purified by renografin density-gradient centrifugation. Uninfected Vero cells were used as negative antigen controls. Human serum was diluted 1:100 in PBSTM (0.1 mol/L PBS, 0.05% Tween 20, and 0.5% nonfat dry milk). Alkaline phosphatase–labeled donkey antihuman IgG was used as a secondary antibody at a 1:10,000 dilution in PBSTM. BCIP-NBT (5-bromo-4-chloro-3-indolyl phosphate-nitro blue tetrazolium chloride) was used as the substrate for alkaline phosphatase and color development. The assay was validated by using the same positive and negative controls used in the IFA.
